# Path Following Control for Underactuated Airships with Magnitude and Rate Saturation

**DOI:** 10.3390/s20247176

**Published:** 2020-12-15

**Authors:** Huabei Gou, Xiao Guo, Wenjie Lou, Jiajun Ou, Jiace Yuan

**Affiliations:** 1 School of Aeronautic Science and Engineering, Beijing University of Aeronautics and Astronautics, Beijing 100191, China; ghbuaaa@buaa.edu.cn (H.G.); oujiajun@buaa.edu.cn (J.O.); yuanjiace@buaa.edu.cn (J.Y.); 2 Frontier Institute of Science and Technology Innovation, Beijing University of Aeronautics and Astronautics, Beijing 100191, China; guoxiao@buaa.edu.cn; 3 School of Electronic and Information Engineering, Beijing University of Aeronautics and Astronautics, Beijing 100191, China

**Keywords:** reinforcement learning, path following, underactuated airships, magnitude and rate saturation

## Abstract

This paper proposes a reinforcement learning (RL) based path following strategy for underactuated airships with magnitude and rate saturation. The Markov decision process (MDP) model for the control problem is established. Then an error bounded line-of-sight (LOS) guidance law is investigated to restrain the state space. Subsequently, a proximal policy optimization (PPO) algorithm is employed to approximate the optimal action policy through trial and error. Since the optimal action policy is generated from the action space, the magnitude and rate saturation can be avoided. The simulation results, involving circular, general, broken-line, and anti-wind path following tasks, demonstrate that the proposed control scheme can transfer to new tasks without adaptation, and possesses satisfying real-time performance and robustness.

## 1. Introduction

As a kind of lighter-than-air vehicles, airships have distinct advantages over other vehicles in ultra-long duration flight, low fuel consumption, making them a cost-effective platform for communication relay, monitoring, surveillance, and scientific exploration. Many countries regard airships as one of the most important platforms in near space and have developed techniques about airships for decades [1,2,3].

With the rapid progress of airship technologies, the essential role of flight control is more prominent. Path following is one of the most frequently performed tasks in flight control, which requires the airship to follow a predefined geometric path without temporal constraint. Due to the inherent dynamics nonlinearity, unknown dynamics, parametric uncertainty, and external disturbance, airship path following control becomes a challenging research topic [4,5]. Numerous achievements on the path following problems have been witnessed, employing various methods such as sliding mode control (SMC) [6,7], H-infinity [8,9], backstepping control [4,10,11,12], fuzzy control [13,14,15,16,17,18], and so forth. In Reference [19], a computationally efficient observer-based model predictive control (MPC) method is presented to achieve arbitrary path following for marine vessels. In Reference [20], a path following controller for a quadrotor with a cable suspended is proposed. The coordinated path following control for multiple nonlinear autonomous vehicles connected by digital networks is studied in Reference [21]. In Reference [22], the authors investigate the reliable H-infinity path following controller for an autonomous ground vehicle.

Although various studies about path following control are investigated, there are still many challenges and obstacles for viable implementation. One of the challenges is the actuators’ magnitude and rate saturation, which is ubiquitous in engineering. For airships, the magnitude saturation is the upper and lower limits of the thrust and the rudder deflection, while the rate saturation is the corresponding acceleration saturation. The saturation problem normally means the amplitude and rate constraints of the input signals during the controller design. Once the constraints are violated, it may lead to performance deterioration or system instability [23,24]. Various control strategies are investigated to handle the saturation problem [25,26,27,28,29]. In Reference [30], a backstepping controller combined with auxiliary systems is designed to meet the input magnitude and rate constraints for a two-blade propeller system of an aircraft. In Reference [31], a decentralized state feedback controller based on the linear matrix inequality (LMI) conditions is adopted to a class of nonlinear interconnected systems subject to the magnitude and rate constraints. In Reference [32], the LMI and Lyapunov direct techniques are combined to deal with the input magnitude and rate saturation for a flexible spacecraft. In Reference [33], the backstepping scheme with smooth hyperbolic tangent function is proposed to address the magnitude and rate saturation for the nonlinear three-dimensional Euler-Bernoulli beam. However, the problem has not been fully studied in the domain of airship path following control. Due to the complexity of the problem, it is difficult to implement Lyapunov based methods. MPC is served as an effective solution by considering control problem as a process of optimization, but heavy computing burden hampers the real-time implementation.

Recently, reinforcement learning (RL) has been successfully applied in robot control, underwater vehicle guidance, and path planning [34,35,36]. Owing to the performance of model insensitivity, RL can achieve control objectives for various complex dynamic systems. Besides, RL becomes so compelling for its prominent advantage in treating optimization problems. Therefore, it is feasible to solve the airships’ magnitude and rate saturation problems from the perspective of optimization with RL strategies. Generally, RL methods can be divided into two categories—policy search methods and value-based methods. For the policy search methods, the action-selection policies are explicit rather than implicitly derived from separate value functions, which makes it more efficient than value-based methods when dealing with the continued-states and high-dimension problems [37,38]. There are several popular RL algorithms such as trust region policy optimization (TRPO) [39], proximal policy optimization (PPO) [40], soft actor-critic (SAC) [41], and twin delayed deep deterministic policy gradient (TD3) [42]. Considering the efficiency and stability, the PPO algorithm is adopted in our approach, which is one of the state-of-the-art learning algorithms.

With the enrichment of application scenarios, the controller of airships must have the ability to adapt to a variety of tasks. There are two challenges that must be considered during the controller design process [43]. First, transferring policies proves difficult on task distributions for the optimal policy varies dramatically across tasks. Second, transferring policies affects the time and sample efficiency in the adaptation phase. Motivated by the discussions mentioned above, the RL-based path following controller is proposed for underactuated airships subject to magnitude and rate saturation in this paper. To realize real-time performance and transfer action policy between tasks, an error bounded LOS guidance law is presented to restrain the state space, while a PPO-based algorithm is employed to acquire the action policy through trial and error. Frequently performed tasks are converted to a point-following task while the point is always distributed in a specified bounded space. Exhaustive training in this space will generate optimal action policies.

Contributions of this paper are as follows:

- An RL-based path following controller is proposed for underactuated airships with magnitude and rate saturation. Compared with the nonlinear MPC method referenced from Reference [44], the proposed method has better performance in real-time and comparable control quality.
- An error bounded LOS guidance law is presented to provide task-independent state space, enabling the trained controller to transfer to new tasks without adaptation. Besides, a PPO-based algorithm is employed to generate the action policy through trial and error.
- The capability of transferring between tasks is demonstrated by performing circular, general, and broken-line path following tasks, while the robustness is proved by circular path following under wind disturbance.

The rest of this paper is organized as follows: Section 2 presents the airship model. The design of the controller is shown in Section 3. Section 4 simulates the proposed controller. Discussion and conclusion are given in Section 5 and Section 6, respectively.

## 2. Airship Model

**Figure 1:**
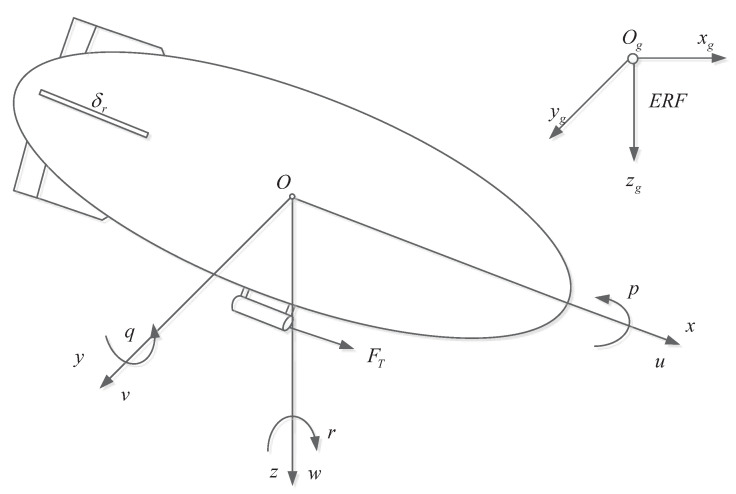
Structure and coordinate systems of the airship.

The airship studied in this paper is depicted in Figure 1. The static lift of the airship is provided by the helium contained in the envelope. The aerodynamic control surfaces attached to the rear of the airship, namely the tails and rudders, offer the control torques for the airship and enhance the course stability. The propellers are mounted symmetrically on the sidewall of the gondola fixed at the bottom of the envelope.

To develop the airship model, the body reference frame (BRF) is firstly defined. The BRF is attached to the airship with its origin *O* coincident with the center of volume. Ox points towards the head of the airship, Oy is perpendicular to Ox towards the right of the airship, and Oz is determined by the right-hand rule. The origin $O_{g}$ of the earth reference frame (ERF) is fixed on the ground, $O_{g}x_{g}$ and $O_{g}y_{g}$ point to the north and east, respectively. $O_{g}z_{g}$ is determined by the right-hand rule.

According to Reference [45], the model of the airship is described as:

(1) $$\left[\begin{array}{c} \dot{\zeta }\\ \dot{\Theta } \end{array}\right]=\left[\begin{array}{cc} K & 0_{3\times 3}\\ 0_{3\times 3} & R \end{array}\right]\left[\begin{array}{c} v\\ \Omega \end{array}\right]$$

(2) $$\begin{array}{c} \bar{A}\left[\begin{array}{c} \dot{v}\\ \dot{\Omega } \end{array}\right]=\bar{N}+\bar{G}+\bar{B}\left[\begin{array}{c} u_{F}\\ u_{\delta } \end{array}\right], \end{array}$$

where $\zeta ={\left[x,y,z\right]}^{T}$ and $\Theta ={\left[\phi ,\theta ,\psi \right]}^{T}$ are the position and attitude of airship described in ERF, respectively. $v={\left[u,v,w\right]}^{T}$ and $\Omega ={\left[p,q,r\right]}^{T}$ are velocity and angular velocity described in BRF, respectively. The detailed expressions of $\bar{A}$, $\bar{N}$, $\bar{G}$, $\bar{B}$, $u_{F}$ and $u_{\delta }$ are described in Reference [45].

To facilitate the design of the controller, the attitude motion of pitch and roll in the vertical plane is ignored and only the horizontal motion is considered in this study [9]. Thus, the values of the variables w,p,q,ϕ,θ, and *z* are set as zero. Consequently, the planar linear kinematics and dynamic state-space equations can be derived from (1) and (2) as:

(3) $$\left[\begin{array}{c} \dot{\psi }\\ \dot{x}\\ \dot{y} \end{array}\right]=\left[\begin{array}{ccc} 1 & 0 & 0\\ 0 & \cos\psi & -\sin\psi \\ 0 & \sin\psi & \cos\psi \end{array}\right]\left[\begin{array}{c} r\\ u\\ v \end{array}\right]$$

(4) $$\left\{\begin{array}{c} {\dot{X}}_{1}=AX_{1}+BF\\ {\dot{X}}_{2}=CX_{1}+DF\\ , \end{array}\right.$$

where $X_{1}={\left[r,u,v\right]}^{T}$ is the state, $X_{2}={\left[\psi ,x,y\right]}^{T}$ is the measured output, and $F={\left[F_{T},\delta_{r}\right]}^{T}$ is the control input, where $F_{T}$ denotes the thrust force and $\delta_{r}$ denotes the rudder deflection. The specific expressions of the variables above are described in Reference [9].

## 3. Path Following Controller Design

**Figure 2:**
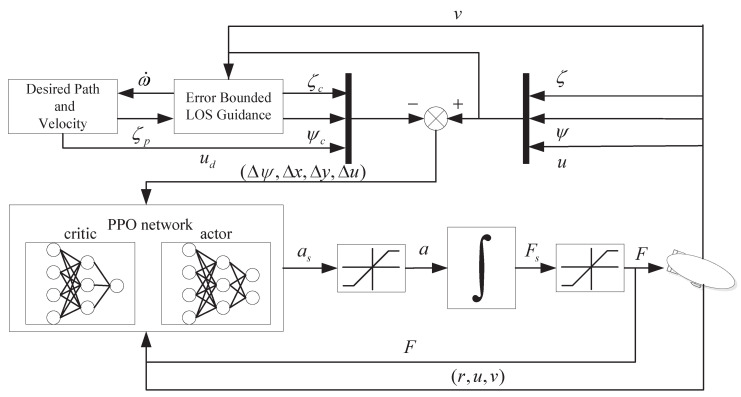
Structure of the proposed path following controller.

In this section, a path following controller is developed for underactuated airships with magnitude and rate saturation. The proposed controller consists of two sub-systems: error bounded LOS guidance and PPO control. The guidance loop provides the desired heading angle and the target position, and PPO calculates the near-optimal solution in bounded space. In PPO control, the Markov decision process (MDP) model is constructed firstly. Then, the reward function is designed based on the MDP model. Finally, the optimization process of PPO is introduced. Figure 2 shows the structure of the controller.

During the design process, the state space is reduced to an acceptable range. After adequate training, most states in the space will be experienced. Consequently, the desired action policy can be obtained, realizing the real-time path following.

### 3.1. Error Bounded LOS Guidance

The underactuated airship is desired to follow a continuous path $\zeta_{p}\left(\omega \right)={\left[x_{p}\left(\omega \right),y_{p}\left(\omega \right)\right]}^{T}$ parameterized by a time-independent variable ω. Suppose that the target position provided by the path is $\zeta_{p}=\left(x_{p},y_{p}\right)$, a path parallel frame (PPF) can be defined: its origin point is $\zeta_{p}$, $\zeta_{p}x_{p}$ is parallel with the local tangent vector, and $\zeta_{p}y_{p}$ is the normal vector pointing to the right. The ERF should be positively rotated by an angle $\psi_{p}$ to reach PPF.

(5) $$\psi_{p}=arctan2\left(\frac{dy_{p}}{d\omega },\frac{dx_{p}}{d\omega }\right).$$

Thus, the tracking error in PPF can be expressed as follows:

(6) $$\epsilon ={\left[s,e\right]}^{T}=R\left(\psi_{p}\right)\left(\zeta -\zeta_{p}\right),$$

where *s* is the along-track error, *e* is the cross-track error, and $R(\psi_{P})$ is given by:

(7) $$R\left(\psi_{P}\right)=\left[\begin{array}{cc} \cos\psi_{P} & \sin\psi_{P}\\ -\sin\psi_{P} & \cos\psi_{P}. \end{array}\right]$$

To reduce the state space, the target position $\zeta_{c}=\left(x_{c},y_{c}\right)$ is selected as follows:

(8) $$\zeta_{c}=\zeta +K_{r}tanh\left(\frac{\zeta_{p}-\zeta }{K_{r}}\right),$$

where $K_{r}>0$. The target position $\zeta_{c}$ will remain in a circle near current position.

(9) $$\zeta -\zeta_{c}=K_{r}tanh\left(\frac{\zeta -\zeta_{p}}{K_{r}}\right)$$

(10) $$K_{r}atanh\left(\frac{\zeta -\zeta_{c}}{K_{r}}\right)=\zeta -\zeta_{p}.$$

Obviously, the tracking error $\zeta -\zeta_{p}$ will converge to zero as $\zeta -\zeta_{c}$ converge to zero. Considering Lyapunov function as follows:

(11) $$V_{\epsilon }=\frac{1}{2}{K_{r}}^{2}{\left(R\left(\psi_{p}\right)atanh\left(\frac{\zeta -\zeta_{c}}{K_{r}}\right)\right)}^{T}R\left(\psi_{p}\right)atanh\left(\frac{\zeta -\zeta_{c}}{K_{r}}\right).$$

Substituting Equation (10) into Equation (11):

(12) $$\begin{array}{cc} V_{\epsilon } & =\frac{1}{2}{K_{r}}^{2}{\left(R\left(\psi_{p}\right)atanh\left(\frac{\zeta -\zeta_{c}}{K_{r}}\right)\right)}^{T}R\left(\psi_{p}\right)atanh\left(\frac{\zeta -\zeta_{c}}{K_{r}}\right)\\ \; & =\frac{1}{2}{\left(R\left(\psi_{p}\right)\left(\zeta -\zeta_{p}\right)\right)}^{T}\left(R\left(\psi_{p}\right)\left(\zeta -\zeta_{p}\right)\right)\\ \; & =\frac{1}{2}\epsilon^{T}\epsilon . \end{array}$$

Differentiating Equation (12) with respect to time, it leads to:

(13) $$\begin{array}{cc} {\dot{V}}_{\epsilon } & =\epsilon^{T}\dot{\epsilon }\\ & =\epsilon^{T}\left(\dot{R}\left(\psi_{p}\right)\left(\zeta -\zeta_{p}\right)+R\left(\psi_{p}\right)\left(\dot{\zeta }-{\dot{\zeta }}_{p}\right)\right)\\ & =s(u\cos\left(\psi -\psi_{p}\right)-v\sin\left(\psi -\psi_{p}\right)-\dot{\omega }\sqrt{{\left(\frac{dx_{p}}{d\omega }\right)}^{2}+{\left(\frac{dx_{p}}{d\omega }\right)}^{2}}+e(u\sin\left(\psi -\psi_{p}\right)+v\cos\left(\psi -\psi_{p}\right). \end{array}$$

Designing ψ and $\dot{\omega }$ as follows [46]:

(14) $$\begin{array}{cc} \psi =\psi_{c} & =\psi_{p}+\psi_{r}-\beta_{d}\\ \; & =\psi_{p}+arctan2\left(-e,k_{e}\right)-arctan2\left(v,u\right) \end{array}$$

(15) $$\dot{\omega }=\frac{u_{d}\cos\left(\psi -\psi_{p}\right)-v\sin\left(\psi -\psi_{p}\right)+k_{s}s}{\sqrt{{\left(\frac{dx_{p}}{d\omega }\right)}^{2}+{\left(\frac{dx_{p}}{d\omega }\right)}^{2}}},$$

where $k_{e}>0$ and $k_{s}>0$. Substituting Equation (14) and Equation (15) into Equation (13) yields:

(16) $${\dot{V}}_{\epsilon }=-k_{s}s^{2}-\frac{u\sqrt{u^{2}+v^{2}}}{\left|u\right|\sqrt{e^{2}+{\left(k_{e}\right)}^{2}}}e^{2}=-k_{s}s^{2}-\frac{\sqrt{u^{2}+v^{2}}}{\sqrt{e^{2}+{\left(k_{e}\right)}^{2}}}e^{2}\leq 0\left(ifu\geq 0\right).$$

According to the derivation above, the desired yaw angle ψ and the derivative of the path parameter $\dot{\omega }$ are obtained. Since the vertical plane motion is not considered, the desired attitude $\Theta_{c}$ is also obtained.

### 3.2. MDP Model of the Airship

MDP is a discrete-time stochastic control process, providing a mathematical framework for optimization problems solved via RL. MDP can be expressed by a tuple of four elements (S,A,P,R). In time step *t*, the state of the agent is $S_{t}$, and the controller chooses an action $A_{t}$ based on $S_{t}$, independent of all previous states and actions. In next time step t+1, the agent moves to state $S_{t+1}$ and gives the controller an immediate reward *R*, according to the state transition probability *P*. The airships’ MDP model is described as follows.

First, state space *S*. A qualifying state should contain sufficient information for the controller to make reasonable decisions, and avoid invalid information to accelerate the optimization process. In the path following control scenario, the state space *S* is selected as follows:

(17) $$S=\left\{s_{1},s_{2},s_{3},\cdots ,s_{t},\cdots \right\},s_{t}=\left(r,u,v,\Delta \psi ,\Delta x,\Delta y,\Delta u,F_{T},\delta_{r}\right),$$

where $\Delta \psi =\psi -\psi_{c}$, $\Delta x=x-x_{c}$, and $\Delta y=y-y_{c}$. Considering the inherent feature of airships and the guidance loop, we have a bounded state space, where $r\in [-r_{\max},r_{\max}]$, $u\in [0,u_{\max}]$, $v\in [0,v_{\max}]$, Δψ∈[−π,π], $\Delta x\in (-K_{r},K_{r})$, $\Delta y\in (-K_{r},K_{r})$, $\Delta u\in [-u_{\max},u_{\max}]$, $F_{T}\in \left[-F_{\max},F_{\max}\right]$, and $\delta_{r}\in \left[-\delta_{\max},\delta_{\max}\right]$.

Second, action space *A*. To deal with the magnitude and rate saturation problem, the acceleration of actuators is selected as the continuous action space, as well as the output of the PPO network. $sat_{rate}\left(a\right)$ represents the acceleration saturation while $sat_{magnitude}\left(F\right)$ denotes the control variable saturation.

(18) $$sat_{rate}\left(a_{t}\right)=\left\{\begin{array}{c} a_{\min},a_{t}<a_{\min}\\ a_{t},a_{\min}\leq a_{t}\leq a_{\max}\\ a_{\max},a_{t}>a_{\max} \end{array}\right.$$

(19) $$F_{t}=F_{t-1}+\int_{t-\Delta t}^{t}adt$$

(20) $$sat_{magnitude}\left(F_{t}\right)=\left\{\begin{array}{c} F_{\min},F_{t}<F_{\min}\\ F_{t},F_{\min}\leq F_{t}\leq F_{\max}\\ F_{\max},F_{t}>F_{\max}. \end{array}\right.$$

Third, state transition probability *P*. In this study, the state transition is determined by the airship model. In other words, the result of an action in a certain state is unique.

Fourth, reward function *R*. The reward function is described as:

(21) $$\left\{\begin{array}{c} D_{t}=\sqrt{\Delta x^{2}+\Delta y^{2}}\\ R_{t}=k_{D}\left(D_{t}-D_{t-1}\right)-k_{\psi }\left|\Delta \psi \right|-k_{u}\left|\Delta u\right| \end{array}\right.$$

where $k_{D}<0$, $k_{\psi }>0$, and $k_{u}>0$.

### 3.3. Optimization Process of PPO

Policy gradient methods generally consist of a policy gradient estimator and a stochastic gradient ascent algorithm. Usually, the estimator is obtained by differentiating an objective function whose gradient is designed as the policy gradient estimator. In PPO, the clipped objective function [40] is given by:

(22) $$L_{t}^{CLIP+VF+S}\left(\theta \right)={\hat{E}}_{t}\left[L^{CLIP}\left(\theta \right)-c_{1}L_{t}^{VF}\left(\theta \right)+c_{2}S\left[\pi_{\theta }\right]\left(s_{t}\right)\right],$$

where $L_{t}^{VF}\left(\theta \right)={\left(V_{\theta }\left(s_{t}\right)-V_{t}^{targ}\right)}^{2}$, *S* denotes an entropy bonus, and $L^{CLIP}\left(\theta \right)$ is defined as:

(23) $$L^{CLIP}\left(\theta \right)={\hat{E}}_{t}\left[\min\left(r_{t}\left(\theta \right){\hat{A}}_{t},clip\left(r_{t}\left(\theta \right),1-\varepsilon ,1+\varepsilon \right){\hat{A}}_{t}\right)\right],$$

where $r_{t}\left(\theta \right)=\frac{\pi_{\theta }\left(a_{t}|s_{t}\right)}{\pi_{\theta_{old}}\left(a_{t}|s_{t}\right)}$ is the probability ratio, π is the action policy, θ is the policy parameter, and ε is a clip hyperparameter. $clip(r_{t}\left(\theta \right),1-\varepsilon ,1+\varepsilon )$ restricts aggressive policy updates, thus achieving a satisfying performance. ${\hat{A}}_{t}$ is the advantage estimator given by:

(24) $${\hat{A}}_{t}=\delta_{t}+\left(\gamma \lambda \right)\delta_{t+1}+\cdots +{\left(\gamma \lambda \right)}^{T-t+1}\delta_{T-1},$$

where $\delta_{t}=r_{t}+\gamma V\left(s_{t+1}\right)-V\left(s_{t}\right)$, and *T* is a constant much less than the episode length.

The PPO (see Algorithm 1) adopted in this paper includes an actor network and a critic network. As shown in Figure 2, the input layer and hidden layer of the actor and the critic share the same structure. The input states pass through two full connection layers and finally connect to the output layer. The output layer of the actor is a full connection layer with two outputs, representing the probability distribution of accelerations. The output layer of the critic is a full connection layer with one output, representing the state value.
**Algorithm 1** PPO.1:Input: initial actor-network parameter $\theta_{0}$ and critic-network parameter $\phi_{0}$.2: 3:**for** k = 1, 2, ... **do**4: 5:  Run policy $\pi_{\theta_{k}}$ in the environment for T timesteps.6: 7:  Compute rewards-to-go ${\hat{R}}_{t}$.8: 9:  Compute advantage estimates ${\hat{A}}_{t}$ based on the current critic network $V_{\phi_{k}}$.10: 11:  Update the actor network by maximizing the clipped objective function Equation (22) via gradient ascent with Adam [47]:12: 13:  $\theta_{k+1}=\underset{\theta }{\arg\max}L_{t}^{CLIP+VF+S}\left(\theta \right)$14: 15:  Update the critic network via gradient descent algorithm:16: 17:  $\phi_{k+1}=\underset{\phi }{\arg\max}{\left(V_{\phi }-{\hat{R}}_{t}\right)}^{2}$18: **end for**19: 

During training, the processed states and target of the airship are taken as the input of the neural network. The actor-network outputs the derivation of the airship input based on the processed states, while the critic network learns a value function as the actor update basis. The output of the neural network is the acceleration of the control input. After saturation and integration, the control torque and force are obtained.

## 4. Simulations

In this section, five sets of simulations are presented. In the first simulation, the controller is trained by following a circular arc path. To evaluate the performance of the well-trained controller, a comparative path following controller based on nonlinear MPC is introduced, and some simulations are implemented. Subsequently, the performance of the proposed controller is evaluated by following the circular, general, and broken line paths without any adjustment in the next three simulations, respectively. The last simulation is conducted under wind disturbance.

### 4.1. Controller Training and Comparing

During training, the initial states of the airship are $\zeta_{0}={\left[-30\;m,-1530\;m\right]}^{T}$, $\psi_{0}=2\pi \left(rand\left(\right)-0.5\right)\mathrm{rad}$, $r_{0}=0\;\mathrm{rad}/s$, $u_{0}=5\;m/s$, $v_{0}=0\;m/s$. rand() is a random function on the open interval (0,1) generating uniformly distributed pseudorandom numbers. The desired path is given as:

(25) $$\zeta_{p}\left(\omega \right)={\left[1500\sin\left(3.0\omega /1500-\pi \right),1500\cos\left(3.0\omega /1500-\pi \right)\right]}^{T}$$

The physical parameters of the airship are the same as those in Reference [9]. Some of the parameters of the airship and the controller are list in Table 1. After 15,000 episodes of training, the episode reward of the proposed method converges to 15 on average from 150.

As a comparison, an extended nonlinear MPC algorithm for time-varying reference in Reference [44] is introduced. The simulations are performed on an 8-core machine with an Intel Core i5-9300H CPU at 2.40 GHz and 16 GB of RAM. The detailed expression of the algorithm is presented in Algorithm 3.11, Chapter 3 in Reference [44].

**Figure 3:**
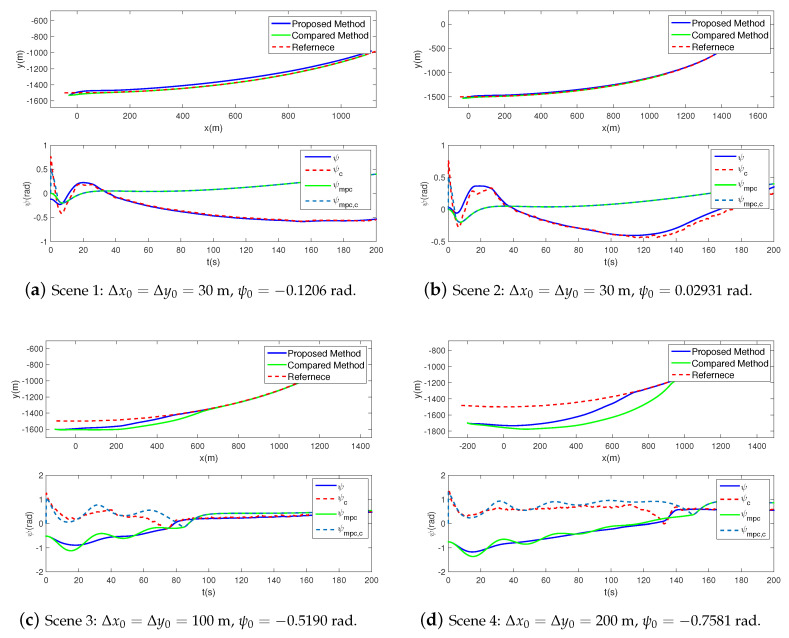
Tracking trajectories and time histories of yaw angles with different initial states for the proposed and the compared controllers.

To estimate the performance of the two controllers, we firstly perform four scenes with different initial states under the same task. The simulation time is set as 200 s, the time step is 0.1 s, and the simulation results are shown in Table 2 and Figure 3. It is obvious in Table 2 that the time consumption of the compared MPC method, averaging 206.63 s, is far greater than the proposed method. That’s even longer than the simulation time, which is unacceptable for practical application. In contrast, the proposed method takes only an average of 1.15 s, demonstrating a satisfying real-time performance in the path-following process.

As shown in Figure 3, the tracking trajectories and the yaw angles are illustrated. It must be emphasized that the desired yaw angles of the proposed and the compared controllers are inconsistent. The reason is that the desired yaw angles are related to the state variables, such as the position and velocity. Due to the differences between the algorithms, different control inputs are obtained, leading to different state variables as well as the desired yaw angles.

For both methods, the trajectories and the yaw angles are convergent in all four scenes. Besides, the greater the initial state differences are, the slower the convergence speeds of the trajectory and the yaw angle get. The distinctions lie that the tracking errors after convergence of the proposed method are 10~30 m, while they are less than 1m of the compared method. The yaw angle error of the proposed method is $10^{-1}$~$10^{-2}$ rad, while it is less than $10^{-3}$ rad of the compared method. Furthermore, the convergence speed of the proposed method is faster than the compared, which is embodied as the trajectories and yaw angles approaching the desired curves with less time in the illustrations. In a word, the proposed method explicates faster convergence speed but worse in tracking precision.

**Figure 4:**
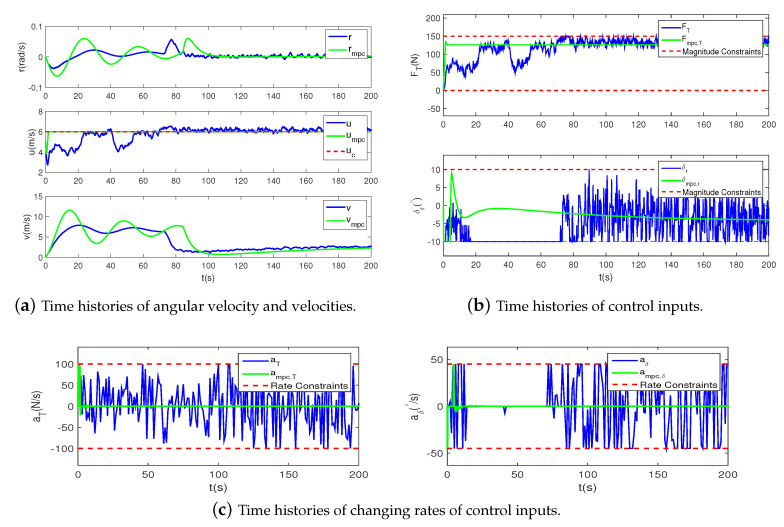
Airship states in Scene 3.

To verify if the magnitude and rate saturation is solved, the state variables of the two controllers in Scene 3 are shown in Figure 4. The simulation time is set as 200 s, and the time step is 0.1 s. Figure 4a shows the time histories of angular velocity and velocities, illustrating that the forward velocity *u* can track its desired value well. Although no desired values for *r* and *v* are set, they are convergent and bounded. Figure 4b gives the time histories of control inputs $F_{T}$ and $\delta_{r}$ with respect to time. Due to actuator rate constraints, the control inputs avoid an initial large jump in References [4,12], which is less harsh to the actuators. In addition, as can be seen from Figure 4b,c, the saturation constraints are never violated. However, the oscillation of the control inputs of the proposed method is relatively serious. Because the execution of the action policies is related to a probability density function, the control inputs are not always continuous.

**Figure 5:**
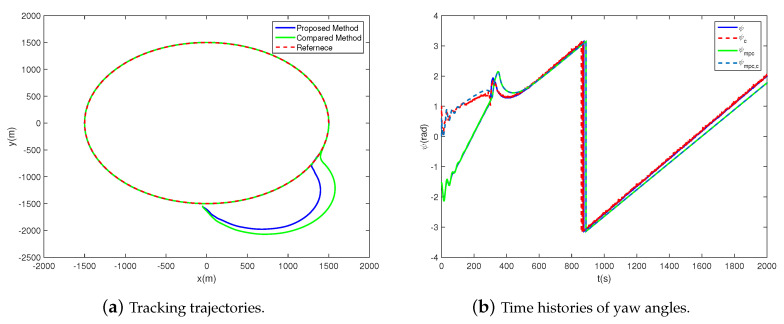

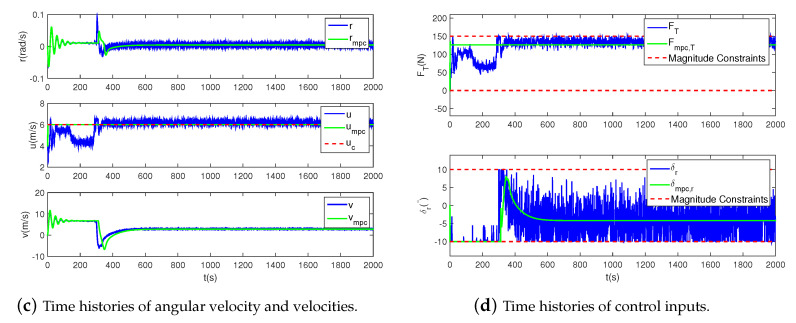
Scene 5: Path following with strong saturation. Initial states: $\Delta x_{0}=\Delta y_{0}=50$ m, $\psi_{0}=-1.5280$ rad.

In extreme circumstances where the airship is desired to follow a path with big initial tracking errors, strong saturation is inevitable. To compare the performance of the two controllers in such situations, a numerical simulation is presented in Figure 5. The time is set as 2000 s, and the time step is 0.1 s. The initial states are chosen as $\Delta x_{0}=\Delta y_{0}=50$ m and $\psi_{0}=-1.5280$ rad. For both controllers, the convergence speeds are much slower than Scene 1–4, consuming more than 300 s. To adjust ψ, the rudder keeps saturated for almost 300 s. During the adjustment, the magnitude and rate constraints are never violated. Besides, compared to the MPC method, the convergence speed of the proposed method is faster while the control inputs and states are less stable.

Conclusions can be drawn from Figure 3, Figure 4 and Figure 5 that the airships can track the desired path subject to actuators’ magnitude and rate saturation under either of the two controllers. Compared with the MPC method, the proposed one is faster in convergence speed but worse in tracking precision, and stronger in the oscillation of the control inputs. Meanwhile, the proposed method has a satisfying real-time performance which the compared method does not. Despite the tracking errors which are acceptable for the airship, the proposed controller has satisfactory effectiveness and robustness in the presence of actuators’ magnitude and rate saturation.

### 4.2. Circular Path Following

**Figure 6:**
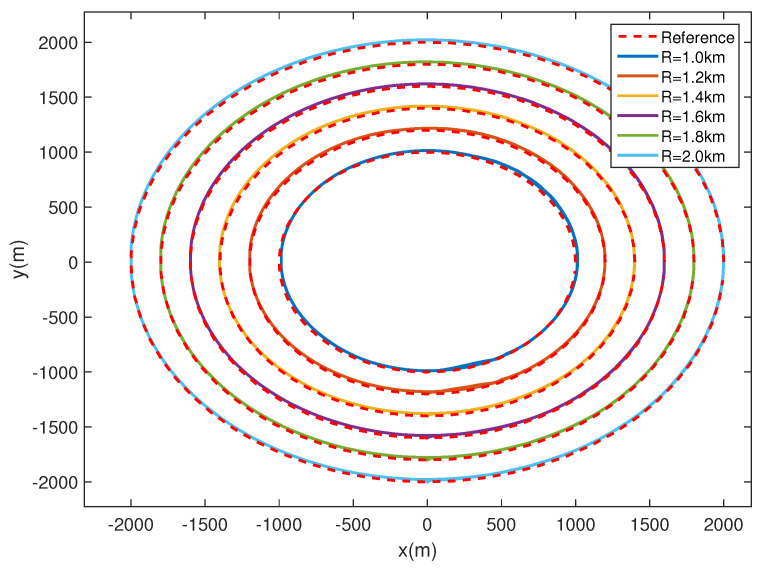
Trajectories of following the circle paths with different radii.

**Figure 7:**
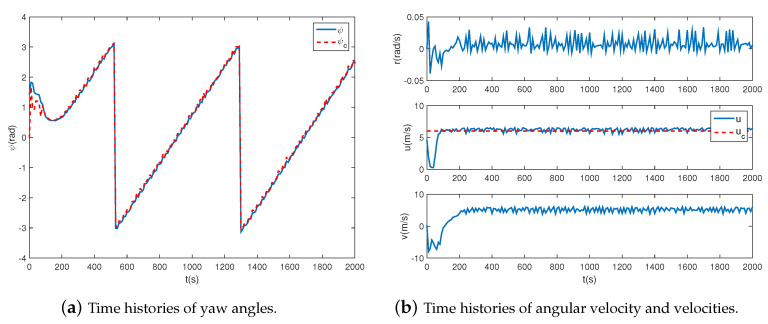
Airship states when R=1.0 km.

**Figure 8:**
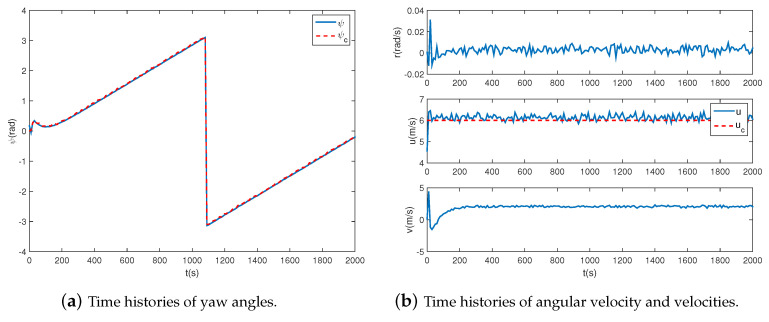
Airship states when R=2.0 km.

In this section, to validate the ability to transfer to new tasks, circular paths with different radii varying from 1 km to 2 km are adopt to the proposed controller. The simulation results are shown in Figure 6, Figure 7 and Figure 8. As shown in Figure 6, the airship is capable of following all circular paths in the presence of magnitude and rate saturation. The average tracking errors are roughly less than 20 m, which implies the task-independent feature of the proposed controller. From Figure 7a and Figure 8a, we learn the real yaw angle is quickly convergent to the desired yaw angle and remain stable. Besides, from the cycle of the airship yaw angles, we learn that the airship takes about 800 s to fly one round with a radius of 1 km, and 2000 s with a radius of 2 km, which implies that the airship has a larger speed when flying around a smaller circle. From Figure 7b and Figure 8b, we learn that both of the desired forward speed *u* is 6 m/s, but the slide speed *v* is different. The slide speed is adjusted bigger to achieve a smaller turning radius. Thus, when flying a circle with a smaller radius, the same forward speed indicates the bigger resultant velocity, leading to a shorter endurance. We also learn that the velocity is tracked well, and the states are stable during path following tasks. It is worth emphasizing that the controller is directly obtained from Section 4.1 without adjustments, which proves the effectiveness of the proposed controller.

### 4.3. General Path Following

**Figure 9:**
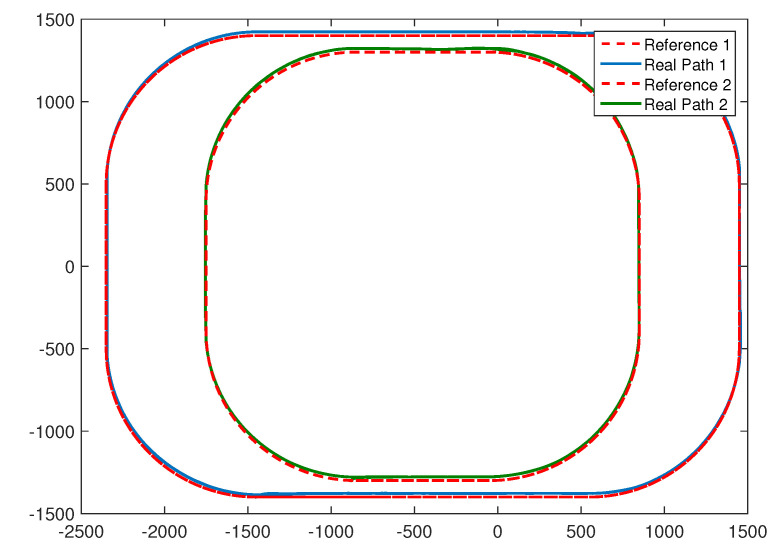
Trajectories of following the general paths with different arcs and line segments.

**Figure 10:**
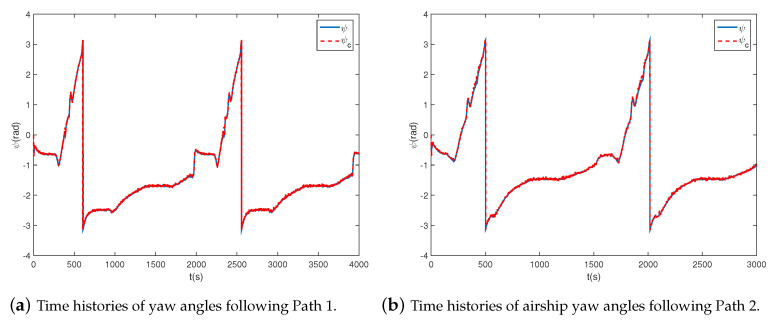
Time histories of yaw angles of the general paths.

This section presents the results of the airship following the general paths of the proposed controller. As shown in Figure 9 and Figure 10, two referenced paths composed of four linear segments and four arc segments are investigated. As can be seen from Figure 9 and Figure 10, the controller accomplishes the tracking tasks well when encountering more general paths. Position errors are reduced efficiently and quickly to an acceptable region. Despite the errors between the referenced and real paths, the tracking states are quite stable.

### 4.4. Broken-line path following

**Figure 11:**
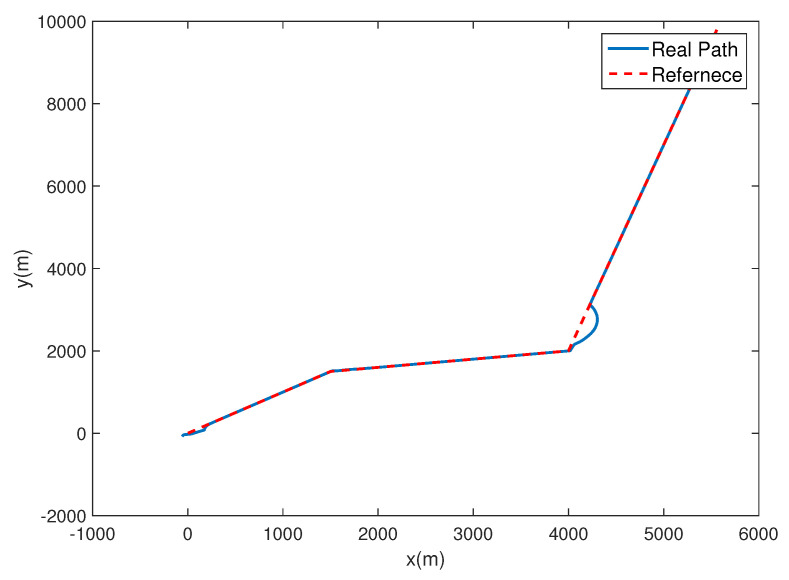
Trajectories of following the broken-line path.

In this section, a simulation of the airship tracking a continuous unsmoothed path is performed. The initial states are chosen as $\Delta x_{0}=\Delta y_{0}=-50$ m and $\psi_{0}=1.1980$ rad. The desired path $\zeta_{p}\left(\omega \right)={\left[x_{p}\left(\omega \right),y_{p}\left(\omega \right)\right]}^{T}$ is a broken-line containing to cusps, which is illustrated in Figure 11, and the parameter equation is given as:

(26) $$\begin{array}{c} x_{p}\left(\omega \right)=\left\{\begin{array}{c} \begin{array}{cc} 3\omega , & 0\leq \omega <50\\ 5\omega -100, & 50\leq \omega <100\\ \omega -300, & 100\leq \omega \leq 200 \end{array} \end{array}\right.\\ y_{p}\left(\omega \right)=\left\{\begin{array}{c} \begin{array}{cc} 3\omega , & 0\leq \omega <50\\ 5\omega -100, & 50\leq \omega <100\\ \omega -300, & 100\leq \omega \leq 200 \end{array} \end{array}\right. \end{array}$$

**Figure 12:**
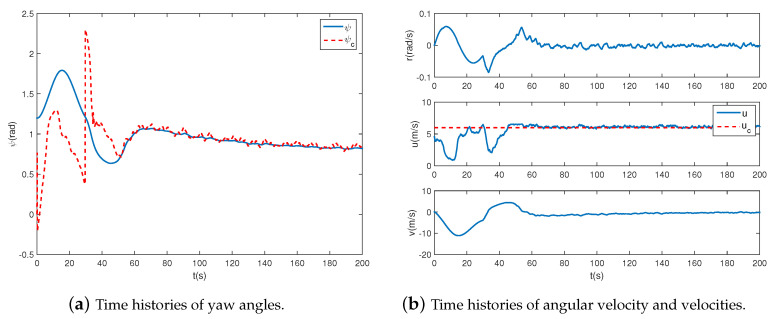
Airship states when following the broken-line path.

As shown in Figure 11, the controller can track the broken-line path well, despite a brief adjustment at the cusp of the path. The tracking error of the yaw angle is quickly convergent to an acceptable region. Besides, Figure 12 indicates that the angular velocity and velocities are relatively stable during the tracking process.

### 4.5. Anti-Wind Path Following

**Figure 13:**
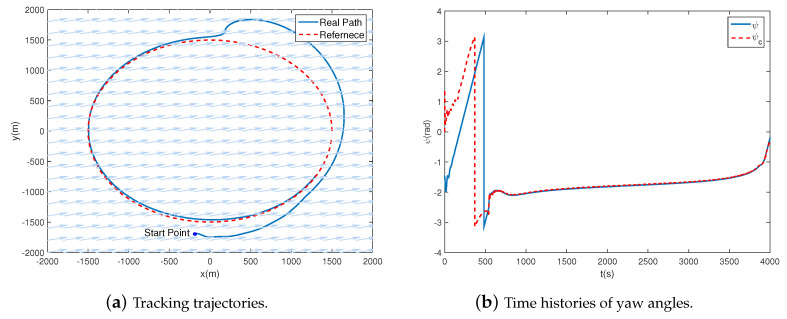
Trajectories of following the path with wind disturbance. Initial states: $\Delta x_{0}=\Delta y_{0}=200$ m, $\psi_{0}=-1.4810$ rad.

In this section, the constant wind disturbance is taken into account when following the desired path. The simulation results are integrated and shown in Figure 13. The initial states are chosen as $\Delta x_{0}=\Delta y_{0}=200$ m and $\psi_{0}=-1.4810$ rad. The wind speed is 5 m/s, as expressed by the light blue arrows in Figure 13a.

Figure 13a illustrates that the airship is capable of following the desired path subject to magnitude and rate saturation, as well as the wind disturbance. Compared with Figure 5a, the airship is forced to cruise for extra miles to converge to the desired path due to the wind disturbance. Figure 13b shows that the yaw angle is well tracked, despite that the airship takes more than 500 s to converge to the desired value.

The simulation results prove the robustness of the controller. Although trained without disturbance, the controller is capable of resisting a wind disturbance.

## 5. Discussion

The proposed RL-based path following controller for underactuated airship shows satisfying performance in numerical simulation. Not only the magnitude and rate saturation of actuators are handled, but the capability of transferring between various tasks without adaption is obtained. Discussions focusing on the two aspects are presented next.

For the target orientation control system, actuators saturation is an inevitable problem. Generally, the actuators saturation problem is handled by auxiliary systems and backstepping technique [28,30,48,49], smooth hyperbolic functions [27,50], model predictive control [9,19,51,52], or employing the generalized nonquadratic cost function [53,54,55] to guarantee the constrained control input. However, the consideration of system capability is always behind the consideration of achieving the control objective. In fact, without first considering the system capability, the designed controllers will be less meaningful in practical application. Unlike the controllers aforementioned, the proposed control strategy is based on building a capacity envelope of the airship. The action policies are generated within the envelope, thus avoiding the saturation. This approach takes full advantage of the adaptivity and robustness of neural networks, more simple to design, and proves to be a feasible solution to the magnitude and rate saturation problems.

On the other hand, transferring to new tasks is a common and challenging problem for RL in practical applications. For airships path following control, the difficulty of transferring to new tasks lies in various task requirements. By reasonably defining the bounded state space *S* and action space *A*, the policies and tasks are decoupled, indicating that the task-independent policies could transfer to new tasks without adaption.

However, there are still some limitations of the proposed method. First, the oscillation of the control input signals is relatively serious, which indicates a high-frequency actuation of the actuators, and will limit the practical implementation of the controller from shortening the service life of the actuators, occupying too much communication bandwidth, or consuming too much energy. Thus the proposed method needs further improvement to reduce the oscillation in the future. Second, the tracking precision of our approach is relatively low. On one hand, the defect in tracking accuracy is caused by the imperfect design of the reward function, which is considerably related to the algorithm accuracy. On the other, the robustness of the RL algorithm is acquired at the expense of tracking precision. There is no specific measurement to counteract the effect of disturbances. Therefore, although the episode reward converges to 10% of the beginning, there’s still room for improvement.

In the future, the oscillation of the control inputs will be reduced. Besides, the tracking precision of our algorithm will be improved. To reach that point, the design of the reward function might be optimized by applying inverse RL methods, and the disturbance will be further considered by employing active observation or compensation. Furthermore, the way of applying the RL strategy to more complex scenarios like spatial flight will be researched.

To sum up, it is a meaningful attempt to combine the policy search strategy with the path following controller. It can be applied to not only airships but also other dynamic systems like vessels, spacecraft, and so forth [19,32,35,48], indicating the broad prospects of the approach. Moreover, the controller can transfer to various tasks without adaption, and resist wind disturbance without anti-disturbance training, demonstrating the research potential of the RL method applying to the flight control field.

## 6. Conclusions

In this paper, a real-time path following controller has been presented for airships with actuator magnitude and rate saturation. First of all, the MDP model of the airship path following control is established, and the error bounded LOS guidance is proposed to restrain the state space. Next, a PPO algorithm containing an actor network and a critic network is utilized to acquire the optimal action policy through trial and error. Since the policy is obtained from the action space, the magnitude and rate saturation will not happen. Finally, the numerical simulation results show that the controller can track circle paths with different radii, general paths, and unsmoothed curves, without any adjustment of parameters. Moreover, even trained without disturbance, the controller shows the satisfying performance when confronting wind disturbance. All these results validate the effectiveness and robustness of the proposed controller. The unique feature of this study lies that the proposed error bounded LOS guidance law provides a task-independent state space for PPO, therefore the trained action policy can transfer to new tasks without adaptation. In the future, the oscillation of the control inputs will be reduced, the tracking precision will be raised by employing anti-disturbance techniques or optimizing the reward function, and RL-based spatial path following control will be studied.

## Figures and Tables

**Table 1:** Parameters of the airship and the controller.

Parameters	Value	Parameters	Value
γ	0.995	λ	0.97
*T*	256	ε	0.2
$F_{\max}$	150	$F_{\min}$	0
$\delta_{r\max}$	10	$\delta_{r_{\min}}$	−10
$a_{F\max}$	100	$a_{F\min}$	−100
$a_{\delta \max}$	45	$a_{\delta \min}$	−45
$k_{e}$	30	$k_{s}$	0.2
$k_{D}$	−0.1	$k_{\psi }$	1.2
$k_{u}$	0.25		

**Table 2:** Time consumption of the proposed and the compared methods.

Time Consumption (s)	Scene 1	Scene 2	Scene 3	Scene 4	Average
proposed method	1.11	1.20	1.12	1.17	1.15
compared method	213.14	199.15	167.92	246.31	206.63
